# Typical metabolic pattern of ^18^F-FDG PET in Anti-NMDAR encephalitis in the acute and subacute phases and its correlation with T2 FLAIR-MRI features

**DOI:** 10.1186/s12868-023-00823-2

**Published:** 2023-09-25

**Authors:** Leilei Yuan, Guangjuan Mao, Yudi Zhang, Yang Xu, Qian Chen, Baoci Shan, Tao Cui, Lin Ai

**Affiliations:** 1 Department of Nuclear Medicine, Beijing Tiantan Hospital, Capital Medical University, Beijing, 100070 China; 2 Beijing Engineering Research Center of Radiographic Techniques and Equipment, Institute of High Energy Physics, Chinese Academy of Sciences, Beijing, 100049 China; 3 School of Nuclear Science and Technology, University of Chinese Academy of Sciences, Beijing, 100049 China; 4 Department of Neurology, The Second Hospital of Hebei Medical University, Shijiazhuang, 050000 China; 5 Department of Neurology, Beijing Tiantan Hospital, Capital Medical University, Beijing, 100070 China; 6 China National Clinical Research Center for Neurological Diseases, Beijing, 100070 China

**Keywords:** Anti-*N*-methyl-D-aspartate receptor encephalitis, NMDA-receptor, ^18^F-FDG PET, Magnetic resonance imaging

## Abstract

**Background/aims:**

Early diagnosis of Anti-N-methyl-D-aspartate receptor (anti-NMDAR) encephalitis with non-invasive imaging modalities benefiting is crucial to guarantee prompt treatments decision-making and good prognosis for patients. The present study aimed to explore the correlation of MRI features with brain metabolism characteristics of ^18^F-fluorodeoxyglucose positron emission tomography (^18^F-FDG PET) and to describe the metabolic patterns in Anti-N-methyl-D-aspartate receptor (anti-NMDAR) encephalitis at acute and subacute phases. Twenty-four patients with anti-NMDAR encephalitis confirmed by serum and/or CSF tests at acute and subacute phases, 9 females and 15 males, with an age range of 6–80 years, were enrolled in this retrospective study as encephalitis group. ^18^F-FDG PET and MRI findings of all patients were investigated and interpreted with visual analysis. Chi-square test was performed to compare the diagnostic sensitivity between MRI and PET. Independent sample *t*-test was used to compare the standardized uptake value ratio (SUVR) of each ROI between the encephalitis group and control group, which consisted of 24 healthy volunteers of the same age and gender.

**Results:**

There was no statistical difference in the diagnostic sensitivity between FDG PET (23/24, 95.83%) and MRI (18/24, 75.00%) in anti-NMDAR encephalitis patients (*P* > 0.05). Three categories of abnormalities shown on T2 FLAIR, including shallow of sulci and swelling of brain tissue, increased signal in the sulci, increased signal on brain gray matter or adjacent white matter presented hypermetabolism on PET, excepting increased signal in brain linear structure with hypometabolism of the basal ganglia on PET. We identified 19 brain regions with hypermetabolism and 16 brain regions with hypometabolism that exhibited statistically significant changes in SUVRs between anti-NMDAR encephalitis group and control group (FDR *P* < 0.05).

**Conclusion:**

Anteroposterior glucose metabolism gradient (frontal-temporal/parietal-occipital) is proved to be a typical pattern of anti-NMDAR encephalitis at the acute and subacute phases in both visual and statistical testing. Interestingly, the pattern is also commonly found in the anterior and posterior portions of the parietal lobe and cingular cortex, which may be a potential indicator for the diagnosis of this disorder. In addition, MRI is an important and reliable neuroimaging modality to assist in the correct evaluation of activity changes on individual ^18^F-FDG PET.

## Background

Anti-N-methyl-D-aspartate receptor (NMDAR) encephalitis is a form of immunological encephalopathy (IE). It is a central nervous system (CNS) autoimmune disorder that is caused by autoantibodies that target the NMDA receptor. The condition result in considerable disability and death [1–3]. Some precipitating factors of anti-NMDAR encephalitis have been identified, which include ovarian teratomas and viral infections, though the triggers remain unknown in many cases. Anti-NMDAR encephalitis has progressed from an uncommon paraneoplastic condition to the most common form of non-viral encephalitis within the previous decade.

Anti-NMDAR encephalitis is caused by an autoantibody against the glutamate receptor N1 (GluN1) subunit of the NMDA receptor. GluN1 is ubiquitously found in the brain but mainly in the frontotemporal areas and the hippocampus. GluN1 can reversibly cause a selective titer-dependent decrease in glutamatergic synaptic function via antibody-mediated capping, cross-linking, and internalization of NMDA receptors [4–6]. The synaptic function reduces without a substantial loss of synapses or other synapse proteins undergird the reversible clinical process. This way, symptoms can be possibly reversed with treatment that is directed against antibodies. Corticosteroids, IVIG, plasma exchange, cyclophosphamide, rituximab, or bortezomib can be used for treatment [7, 8]. Therefore, early diagnosis and treatment are crucial since prognosis largely depends on prompt and adequate immunotherapy and complete tumor resection in paraneoplastic cases. Unfortunately, many studies have reported residual deficits in psychiatric symptoms, memory, speech and cognition in recovered patients [9, 10]. Some clinical red flags for making a differential diagnosis were identified, such as young patients with acute-onset psychotic features, a relatively short course of illness (days to weeks), the triad of either catatonia or seizures plus panic and sleepiness, as well as new onset of seizures on the background of behavioral symptoms. Even so, the variety of clinical manifestations still makes it difficult for neurologists or psychiatrists, especially those who are not familiar with this disorder, to make a correct diagnosis or suggest precise treatment strategies. This scenario results in unpredictable mental and neurological disabilities, to the point of threatening life [1, 11]. Results from a questionnaire study showed that 48.7% of psychiatrists were not aware of the existence of this disorder, 30.3% were only familiar with the name of the disorder, and 21% knew the outline of the disorder. Based on this, more anti-NMDAR encephalitis awareness campaigns among practicing clinicians, especially psychiatrists, are of paramount importance [12]. Another study reported that there was approximately one in eight adults with anti-NMDAR encephalitis inappropriately admitted to the psychiatric unit [13].

The presence of anti-NMDAR antibodies in the serum or cerebrospinal fluid (CSF) can typically provide the most definitive evidence for diagnosing anti-NMDAR encephalitis. However, there are some disadvantages of antibody testing. First, not all hospitals carry out serum or CSF tests, and where they are done, waiting for antibody testing results may delay a definite diagnosis. Second, the absence of antibodies in CSF does not definitively exclude anti-NMDAR encephalitis, considering the role of the brain as a site for antibody immune complex precipitation, which may cause a negative CSF result [14]. Traditional or standard techniques such as EEG and MRI may provide some additional information for a more precise diagnosis. However, the characteristic “extreme delta brush” of EEG findings can be found in only up to one-third of adult cases [15]. Furthermore, the diagnostic sensitivity ranging from 11 to 83% had been reported for MRI, adding to the difficulties associated with diagnosing the condition [3, 16]. At present, the clinical application of functional neuroimaging with ^18^F-fluorodeoxyglucose positron emission tomography (^18^F-FDG PET) sheds light on this challenging problem. Moreover, some previous research demonstrated a more sensitive detection capability of ^18^F-FDG PET compared to MRI [17]. Some characteristic patterns of glucose metabolism were identified to provide important clues to improve decision-making in clinical diagnosis. Bilateral hypometabolism in the occipital lobe is considered a typical metabolic pattern used that helps the physician to start empirical treatment before antibody-positive confirmation. Marked medial occipital lobe hypometabolism may serve as an early biomarker for screening anti–NMDAR encephalitis from other AE [18]. The frontal-temporal-occipital gradient was widely discussed and recognized in various research endeavors [19, 20]. However, there are still many unsolved questions. The metabolism change of the partial lobe is still controversial [21, 22]. Most previous studies or case reports did not explore the correlation between metabolism patterns and disease phases due to the limitations of the small sample size [21]. Some studies took disease stags into account but only covered a small number of patients [22]. It’s also important to note that the correlation between PET findings and abnormalities on MRI was not discussed in sufficient depth.

The present study was designed to illustrate the diagnostic models of initial ^18^F-FDG PET combined with T2 FLAIR-MRI, using a relatively large sample size, and to determine the metabolic pattern of anti-NMDAR encephalitis patients in the acute and subacute phases.

## Methods

### Patients

Twenty-four hospitalized patients with anti-NMDAR encephalitis in Beijing Tiantan Hospital from September 2014 to February 2022 were enrolled in this study. The main inclusion criteria were (1) a confirmed diagnosis of anti-NMDAR encephalitis based on antibody-positive findings in Serum or CSF; (2) ^18^F-FDG PET scans were performed within the acute/subacute phases, which is 3 months from the last onset [23]; (3) completed ^18^F-FDG PET/CT sequences scanning and MRI; and (4) complete and detailed medical records. The exclusion criteria were as follows: (1) patients with brain tumors, cerebral infarction and other brain structural abnormalities; (2) patients with brain operative histories; (3) insufficient quality of FDG PET/CT images for diagnosis and assessment; and (4) any artifacts found on traditional MRI. Twenty-four healthy volunteers of the same age and gender were enrolled as the control group. All volunteers had no neuropsychiatric disorder or abnormalities on neurological examinations, and no abnormal PET findings were found in the brain based on the visual evaluation.

### Clinical data collection

The electronic medical record was reviewed, and clinical data collected included demographic information, clinical symptoms and test results. The clinical symptoms included epileptic seizures, movement disorders, memory impairment, autonomic instability, disturbances of consciousness, and psychiatric symptoms. All the clinical symptoms were determined by a neurologist (Tao Cui) with at least 5 years of clinical practice experience. The administration of corticosteroids or sedatives before the ^18^F-FDG PET was also recorded.

### Imaging acquisition, processing and visual analysis

^***18***^***F-FDG PET imaging acquisition***. ^18^F-FDG PET images were acquired with PET/CT (GE Healthcare, Discovery Elite 670, USA). All patients fasted for 4–6 h before scanning. The pre-injection blood glucose levels were confirmed to be ≤ 8 mmol/L. Patients rested in a quiet, dimly lit room after receiving an intravenous injection of ^18^F-FDG at a dose of 0.10–0.15 mCi/kg until the start of imaging. The brain PET images were acquired in the 3D mode for 10 min, and PET reconstruction was performed with an ordered subset expectation maximization (OSEM). A whole-body (including brain region) ^18^F-FDG PET scan was performed for approximately 30 min. The GE Advanced Workstation 4.6 software package (GE Healthcare) was used to generate the ^18^F-FDG PET images.

***Data processing***. First, all PET images were spatially normalized into the Montreal Neurological Institute (MNI) standard space with a toolbox for spatial normalization of brain PET images (SNBPI) [24]. Afterward, the normalized PET images were smoothed to reduce noise with a Gaussian smoothing kernel of 8mm3 full-width at half maximum (FWHM) using SPM12 (Wellcome Department of Imaging Neuroscience, London, UK). Finally, the 116 regions of interest (ROIs) were extracted for each subject based on the anatomical automatic labeling (AAL) atlas using toolbox SNBPI. The standardized uptake value ratio (SUVR) of each ROI was obtained by dividing the mean value of ROI by the mean of the whole brain grey matter for each subject.

***MRI sequences***. The main MR sequences included 3D T1-weighted brain volume imaging (BRAVO), T2-weighted fase spin echo (FSE), T2 fluid-attenuated inversion recovery (FLAIR) and diffusion-weighted imaging (DWI). For BRAVO, the following parameters were applied: TR/TE = 8/3 ms, TI = 450 ms, 1 mm3 isotropic voxels covering the whole head; For T2WI FSE, the following parameters were applied: TR/TE = 6000/140 ms, slice thickness = 4 mm, slice gap = 1 mm, pixel size = 0.36 × 0.36 mm^2^ ; For FLAIR, the following parameters were applied: TR/TE = 11,000/140 ms, slice thickness = 4 mm, slice gap = 1 mm, pixel size = 0.47 × 0.47 mm^2^; For DWI, the following parameters were applied: TR/TE = 5800/70 ms, slice thickness = 2 mm, slice gap = 0, pixel size = 2 × 2 mm^2^, included two volume at b = 0 s/mm2 and b = 1000 s/mm^2^. Abnormalities on T2 FLAIR were categorized into four types, including shallow of sulci and swelling of brain tissue, increased signal in the sulci, increased signal on brain gray matter or adjacent white matter, and increased signal in brain linear structure or the basal ganglia. In this study, positive MRI findings were defined when one or more indications were presented.

***Visual analysis***. All images of ^18^F-FDG PET and MRI were carefully and independently reviewed by two experienced neuroradiologists (Leilei Yuan & Qian Chen) without knowledge of clinical information. FDG metabolism changes shown on PET and abnormalities shown on T2 FLAIR MRI in the hippocampus, basal ganglia and neocortex were all recorded. If there was any inconsistency in the interpretation of results between the two investigators, the final interpretation would be determined by consensus.

### Statistical analysis

Continuous variables were described using descriptive statistics such as mean and standard deviation. Categorical data were described using frequency and percentage. Chi-square test was performed to compare the diagnostic effectiveness between PET and MRI with GraphPad Prism software (version 8.3.0, GraphPad Software Inc., La Jolla, CA). Two sample *t*-test with SPM12 was used to compare the SUVR of each ROI between the anti-NMDAR encephalitis group and the control group, respectively. *P* < 0.05 was considered to be significant.

## Results

### Patients and clinical characteristics

A total of 24 hospitalized patients with anti-NMDAR encephalitis were enrolled in Beijing Tiantan Hospital, from September 2014 to February 2022. The participants included nine females and 15 males. The median age was 25.50 ± 17.24 years, (IQR,18.00–41.50; range, 6–80 years). The clinical characteristics of the included patients are shown in Table 1. Nine of the patients had antibody-positive findings observed in both the serum and CSF while 15 patients had antibody-positive findings in the CSF. There were 18 patients (75.00%) with epileptic seizures, 13 (54.17%) with memory impairment, 12 (50.00%) with psychotic symptoms, and nine (37.50%) with movement disorders. Ten of the patients had medication treatment within 24 h of their PET/CT, and these included three patients who received gamma globulin and steroids, three who received steroids, two who received gamma globulin, one patient who received sedatives and steroids, and another one who received sedatives. No one had a history of a new cancer diagnosis before the performance of the brain or whole-body PET/CT, and no new malignant tumor was found in 12 patients (12/23, 52.17%) with whole-body ^18^F-FDG PET/CT.

**Table 1:** Clinical characteristics of the patients included in the study

Characteristics	Patients
Gender (M/F)	15/9
Age (years): median ± standard deviation	25.50 ± 17.24
Mean duration of symptoms before presentation (wks)	6.54 ± 3.95
Antibody titer (CSF)	
Weakly positive	4
Positive	10
Strongly positive	10
Primary neuropsychiatric symptoms	
Epileptic seizures	18
Memory impairment	13
Psychiatric symptom	12
Movement disorders	9
Treated with drugs within 24 h before PET/CT	
Steroids and gamma globulin	3
Steroids	3
Gamma globulin	2
Sedative and steroids	1
Sedative	1
Whole-body scanning or brain scanning	
Whole-body scanning	12
Brain scanning	12

### PET and T2 FLAIR-MRI findings with visual analysis

The comparison results between ^18^F-FDG PET and T2 FLAIR-MRI findings are shown in Table 2. The chi-square test result showed that there was no difference in diagnostic sensitivity between FDG PET (23/24, 95.83%) and T2 FLAIR-MRI (18/24, 75.00%) in anti-NMDAR encephalitis patients (*P*>0.05). However, statistical differences were observed in diagnostic sensitivity between FDG PET and every type of T2 FLAIR-MRI (*P* < 0.05).

**Table 2:** Comparison of diagnostic sensitivity between T2 FLAIR-MRI and PET.

		T2 FLAIR-MRI interpretation	PET interpretation	Chi-square test, *P*
Positive findings, n (%)	Shallow of sulci	15 (62.50%)	23 (95.83%)	.004^a^*
	Increased signal in the sulci	11 (45.83%)		.000^a^*
	Increased signal on brain gray matter or white matter	12 (50.00%)		.000^a^*
	Any abnormality on T2 FLAIR	18 (75.00%)		.102^b^

*, *P* < 0.05; ^a^, Pearson Chi-square test; ^b^, Continuity correction

According to the visual investigation summarized in Table 3, hypermetabolism on ^18^F-FDG PET was typically found in the frontal lobes (20 [83.33%] consisting of 8B, 4L, and 8R), temporal lobes (19 [79.17%] consisting of 1B, 9L, and 9R), parietal lobes (11 [45.83%] consisting of 3B, 3L, and 5R), putamen (7 [29.17%] consisting of 5B, 1L, and 1R), hippocampus (5 [20.83%] consists of 1B, 1L, and 3R), occipital lobes (4 [16.67%] consist of 1B, 1L, and 2R), caudate (4 [16.67%] consisting of 3B and 1R), thalamus (3 [12.50%] consist of 1L and 2R), insular lobe (1L, 4.17%) and cerebellum (1L, 4.17%). Hypometabolism was commonly found in the occipital lobes (16 [66.67%] consisting of 14B and 2L), parietal lobes (3 [12.50%] consisting of 1B and 2L), frontal lobes (3 [12.50%] consisting of 1B and 2L) and temporal lobes (3 [12.50%] consisting of 2L and 1R). Crossed cerebellar diaschisis (CCD) was found in 11 patients (45.83%), including 8R and 3L decreased glucose metabolism regions.

**Table 3:** Abnormalities on brain ^18^F-FDG PET/CT from visual inspection

Brain lobes	Hypermetabolism				Hypometabolism			
	B	L	R	Total(%)	B	L	R	Total(%)
FRO	8	4	8	20 (83.33%)	1	2	0	3 (12.50%)
PAR	3	3	5	11 (45.83%)	1	2	0	3 (12.50%)
TMP	1	9	9	19 (79.17%)	0	2	1	3 (12.50%)
OCC	1	1	2	4 (16.67%)	14	2	0	16 (66.67%)
INS	0	1	0	1 (4.17%)	0	0	0	0
HIP	1	1	3	5 (20.83%)	0	0	0	0
CAU	3	0	1	4 (16.67%)	0	0	0	0
PUT	5	1	1	7 (29.17%)	0	0	0	0
THA	0	1	2	3 (12.50%)	0	0	0	0
CER	0	1	0	1 (4.17%)	0	0	0	0

B, bilateral; L, left; R, right

FRO, frontal lobe; TMP, temporal lobe; PAR, parietal lobe; PUT, putamen; HIP, hippocampus; OCC, occipital lobe; CAU, caudate; THA, thalamus; INS, insular lobe; CER, cerebellum

**Fig. 1:**
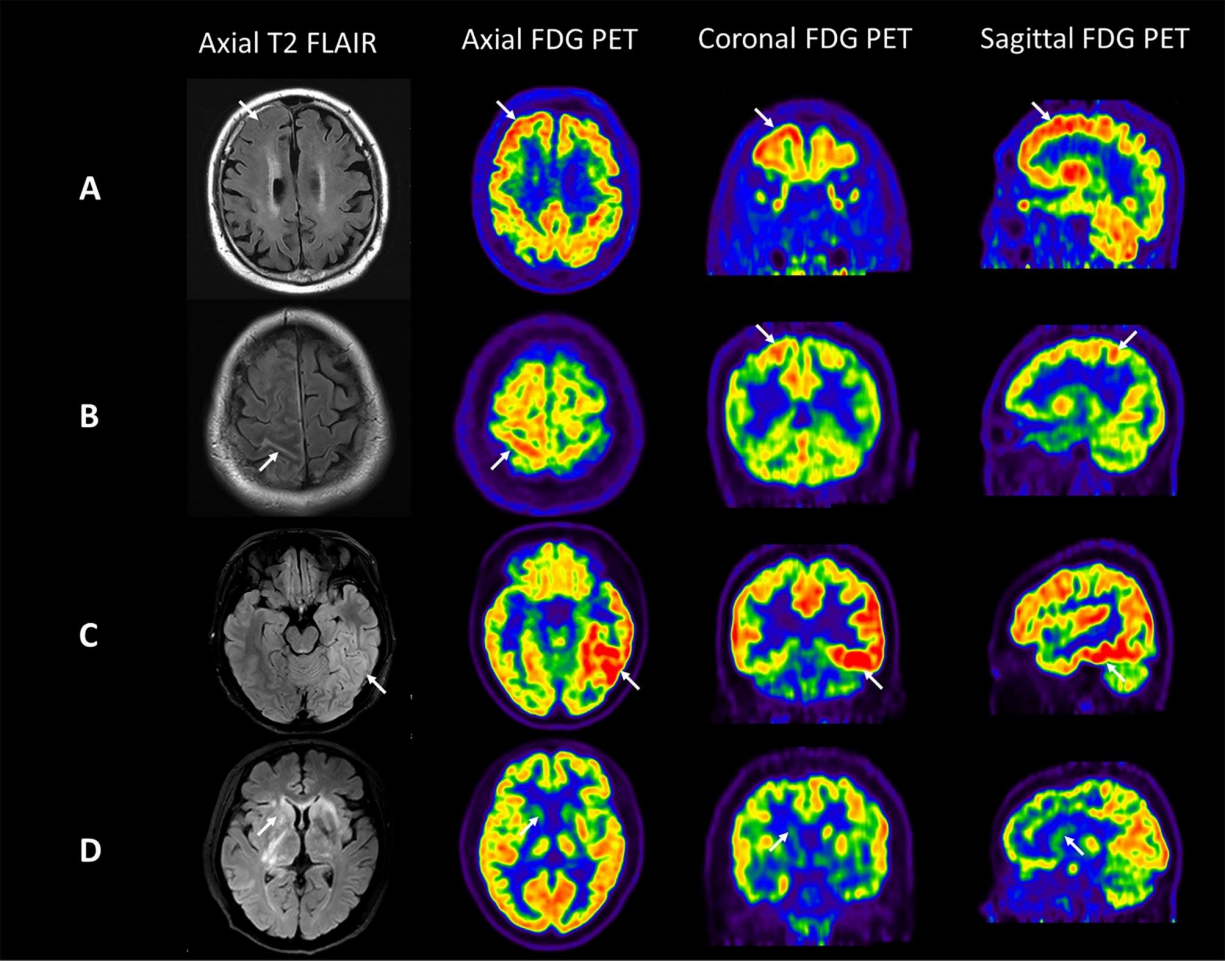
**The correlation of T2 FLAIR findings with**^**18**^** F-FDG PET features in Anti-NMDAR encephalitis patients**. The first row (**A**) shows a 23-year-old woman with a history of psychiatric symptom and epileptic seizures for 9 weeks. Axial T2 FLAIR image shows shallow sulci and brain tissue swelling located in the right frontal lobe. The corresponding axial, coronal and sagittal FDG PET images show an obvious uptake of FDG. The second row (**B**) shows a 31-year-old man with a history of epileptic seizures for 4 weeks. Axial T2 FLAIR image shows increased signal in the sulci of right parietal lobe. The corresponding axial, coronal and sagittal FDG PET images show this linear hypermetabolic lesion. The third row (**C**) shows a 20-year-old woman with a history of psychiatric symptom, memory impairment, epileptic seizures and movement disorders for 3 weeks. Axial T2 FLAIR image shows increased signal on brain gray matter and adjacent white matter of left temporal lobe. The corresponding axial, coronal and sagittal FDG PET images show different degrees of increased metabolism. The last row (**D**) shows a 50-year-old woman with a history of psychiatric symptom, memory impairment and movement disorders for 5 weeks. Axial T2 FLAIR image shows bilateral and diffused increased signal in brain linear structure. The corresponding axial, coronal and sagittal FDG PET images show hypometabolism of basal ganglia

All abnormalities with different characteristics showed on T2 FLAIR-MRI, including shallow sulci and swelling of brain tissue (15/24, 62.50%), increased signals in the sulci (11/24, 45.83%), on brain gray matter or adjacent white matter (12/24, 50.00%), and in brain linear structure or the basal ganglia (2/24, 8.33%). The first three types commonly demonstrated hypermetabolism on ^18^F-FDG PET, whereas brain linear structure and the basal ganglia usually showed hypometabolism (Fig. 1). It’s important to note that several indications referred to above may appear alone or concurrently on MRI. T2 FLAIR images of hypometabolism regions showed no abnormality after retrospective reviews.

### Brain FDG PET characteristics in anti–NMDAR encephalitis patients

**Fig. 2:**
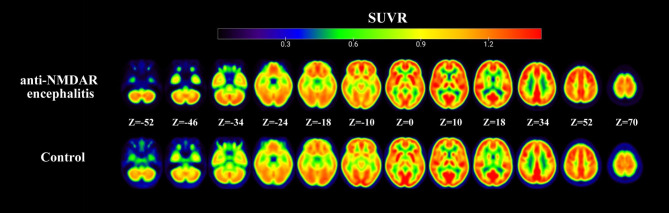
**Mean standardized uptake value ratio (SUVR) images of different groups**. The upper row were SUVR images of the anti–NMDAR encephalitis group (n = 24) and the lower row were SUVR images of control group (n = 24) group

**Fig. 3:**
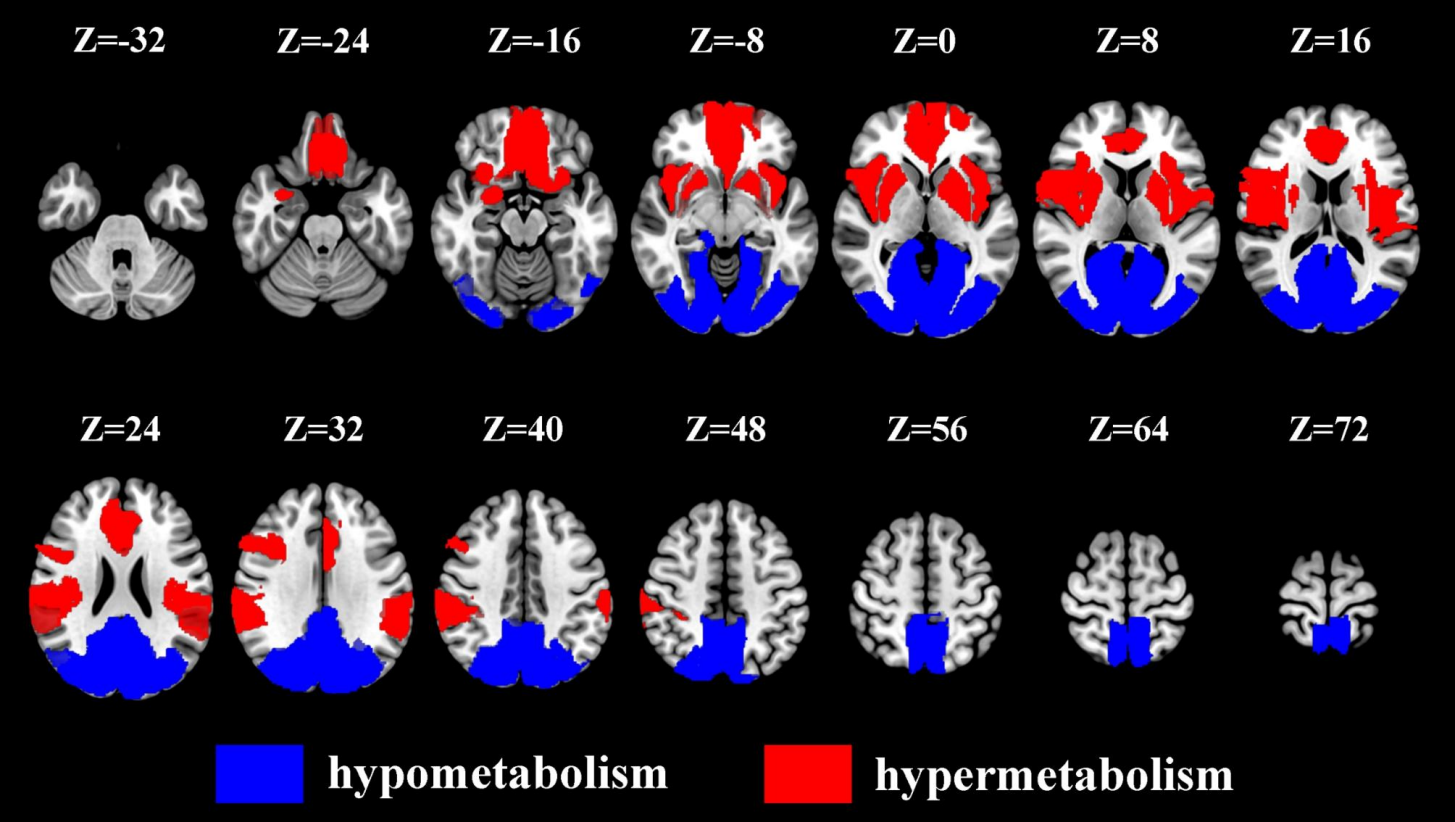
**Glucose metabolic pattern of anti–NMDAR encephalitis patients**. Independent sample t-test was used to compare SUVR) of each ROI between the encephalitis group (n = 24) and control group (n = 24). Thirty-five brain areas with hyper- or hypometabolism of the encephalitis group compared to the control group were depicted with a statistically significant difference (FDR *P* < 0.05), including 19 brain regions with hypermetabolism in red and 16 brain regions with hypometabolism in blue. The T-maps show a typical pattern of anteroposterior glucose metabolism gradient in anti-NMDAR encephalitis patients. SUVR, standardized uptake value ratio; ROI, region of interest

An independent *t*-test was performed to compare the values of 116 brain regions between the anti-NMDAR encephalitis and control groups. Figure 2 showed the mean standardized uptake value ratio (SUVR) images of the anti–NMDAR encephalitis and control groups. Figure 3 depicted 35 brain areas with hyper- or hypometabolism of the encephalitis group compared to the control group, with statistically significant differences (*P*<0.05), including 19 brain regions with hypermetabolism and 16 brain regions with hypometabolism. This result was consistent with the findings from visual analysis, thereby demonstrating focal bilateral hypermetabolism in the frontal, temporal, and anterior parietal lobe as well as bilateral hypometabolism in the posterior parietal lobe and occipital lobe.

## Discussion

This study provides new insights into the typical metabolism pattern of anti-NMDAR encephalitis patients in acute and subacute phases. Much focus was put on revealing the metabolic characteristics of the parietal lobes which have remained controversial. The correlation of ^18^F-FDG PET findings with different T2 FLAIR features was also highlighted to provide objective evidence for early diagnosis. Furthermore, the combination of multiple features of T2 FLAIR-MRI making more patients identified can improve the diagnostic sensitivity of MRI as a first line examination modality, since FDG PET is not available in all hospitals.

The initial symptoms of anti-NMDAR encephalitis range greatly between different studies. Psychiatric symptoms [25], cognitive impairment [26], and seizures [27] are the most common clinical features associated with anti-NMDAR encephalitis. The complexity and variations in clinical symptoms make the diagnosis even more challenging in the initial phases of the disease. The symptoms on patients with anti-NMDAR encephalitis that is mistakenly diagnosed acute psychosis may worsen after treatment with antipsychotics [28, 29]. On the other hand, patients who are misdiagnosed as having degenerative dementia may miss the therapeutic opportunity of reversible cognitive decline of anti-NMDAR encephalitis patients [30]. Therefore, early and accurate diagnosis of anti-NMDAR encephalitis is crucial.

MRI is one of the most commonly used and reliable neuroimaging modalities, which has an important role in excluding differential diagnoses as the first-line method. A systematic review reported that among the assessed 1167 patients in the acute phase, 440 abnormal MRIs (37.7%, 35.0-40.5 95%CI) were found [3]. The present study showed a relatively higher diagnostic capacity of MRI, with a sensitivity of 75.0% in patients who were in the acute and subacute phases. This could be attributed to the current improved understanding of the MRI characteristics of anti-NMDAR encephalitis than before. Otherwise, the presence of any type of abnormal T2 FLAIR findings was considered abnormal MRI, increasing the number of identified patients. According to previous studies, T2 FLAIR hyperintensity was the most commonly reported abnormality [31], and DWI changes were usually accompanied by T2 FLAIR abnormalities, and this was consistent with our research findings. We didn’t find atrophy shown on MRI in these cases, which was previously described as a common sign in a general diffuse pattern. This could be due to the limitation of enrolled patients in the acute and subacute phases. Contrastingly, we found that shallow sulci and swelling of brain tissue, and increased signal in the sulci were commonly observed in the present study, besides increased signal on brain gray matter, adjacent white matter, brain linear structure, or the basal ganglia. If shallow sulci and swelling of brain tissue are regarded as abnormal indicators, the diagnostic sensitivity of MRI can be greatly improved. A sum of 15 patients showed shallow sulci and swelling of brain tissue, and simple brain tissue swelling with no signal abnormalities was observed on three of them. Therefore, we suggested that radiologists and neurologists should consider the possibility of encephalitis as the diagnosis, even when only simple swelling of brain tissue is found on MRI. It should be noted that most of the abnormalities on T2 FLAIR descript above demonstrated hypermetabolism on FDG PET, except alterations of the brain linear structure and basal ganglia on T2 FLAIR with hypometabolism on FDG PET. Although PET didn’t outperform MRI in the current study, considering the higher diagnostic sensitivity (95.83% vs. 75.00%), ^18^F-FDG PET can reveal positive findings in the absence of MRI abnormalities, which have also been reported by previous studies [23].

Nowadays, ^18^F-FDG PET was suggested as a valuable modality in the work-up of suspected anti-NMDAR encephalitis patients, especially in those patients with negative MRI findings. Some characteristic metabolic patterns were identified to provide important clues to improve clinical decision-making. Anteroposterior glucose metabolism gradient was a widely recognized metabolism pattern in this disorder [18–22, 25, 32]. Most research revealed hypermetabolism in frontal and temporal lobes and hypometabolism in bilateral occipital lobes. Different distribution of NMDA receptors, electrophysiology, regulation of both ligand and voltage-gated channels, and varying glucose utilization in different locations of the human brain may contribute to the unknown pathophysiological basis of this metabolic signature of anti-NMDAR encephalitis [33, 34]. The autoantibody against the GluN1 subunit of the NMDA receptor was ubiquitously located in the brain though higher density were reported in frontotemporal areas and the hippocampus [35]. Jing Yuan et al. proposed a hypothesis that abnormal metabolic patterns might be associated with disrupted NMDAR signaling. Ketamine, which is an NMDAR antagonist, showed similar symptoms and frontal-to-occipital gradient of glucose metabolism detected by FDG PET in healthy human participants, as shown in anti-NMDAR encephalitis patients [22].

This present study emphasized this typical metabolism pattern with a relatively large-scale patient number. The cortical visual analysis per patient revealed that frontal lobes were the most susceptible to involvement with hypermetabolism, followed by temporal and parietal lobes. Bilateral hypometabolism in the occipital lobe is considered a typical metabolic pattern that, when present, physicians can start empirical treatment before antibody-positive confirmation. Results from previous studies showed that bilateral decreased metabolism was demonstrated at acute and subacute phases [18, 22], and marked medial occipital lobe hypometabolism may serve as an early biomarker for screening anti-NMDAR encephalitis from other AE [18]. These reports are consistent with our findings. Both visual analysis and “independent *t*-test” demonstrated bilateral occipital hypometabolism in anti-NMDAR encephalitis at acute and subacute phases, which commonly involved bilateral lingual and superior, middle and inferior occipital lobes. Some studies reported the possible association of the glucose hypermetabolism of the frontal and temporal lobes with occipital hypometabolism [20, 22].

We also discovered an interesting phenomenon: when taking unilateral or bilateral affected sites into account, frontal lobes were still the most prone to bilateral involvement with hypermetabolism, but the number of bilateral involved parietal lobes was more than that of the temporal lobes that were involved with hypermetabolism. These were verified by the following cortical SUVR comparison. Hypermetabolism on the anterior portion (supramarginal gyrus) and hypometabolism on the posterior portion (precuneus and cuneus) of the parietal lobes in the anti-NMDAR encephalitis group were proven to be significantly statistically different from the control group, as well as the cingular cortex with hypermetabolism on anterior portion and hypometabolism on posterior portion. This indicated that the parietal lobe and cingular cortex also showed an anteroposterior glucose metabolism gradient. Although previous studies discussed the metabolic change of parietal lobes, its metabolism pattern remained controversial. Many studies reported the hypometabolism of parietal lobes [3, 21, 36, 37], but others demonstrated the hypermetabolism [22]. This research, which involed a relatively large number of patients highlighted that the previously described anteroposterior gradient of activity of the parietal lobe or cingular cortex may be a potential indicator for the diagnosis of anti-NMDAR encephalitis at acute and subacute phases. Therefore, the metabolic changes of the parietal lobe should be investigated and carefully interpreted, instead of simply classifying them as hypometabolism or hypermetabolism. Otherwise, T2 FLAIR-MRI played an important role in determining the hypermetabolism of brain regions or hypometabolism of contralateral brain regions, as correct judgment was sometimes difficult to make. Hypometabolism regions on PET were all negative on T2 FLAIR, but abnormalities on T2 FLAIR were found, indicating hypermetabolism of the corresponding brain regions.

Besides cortical metabolism patterns, some research also revealed the metabolic patterns of basal ganglia nuclei in anti-NMDAR encephalitis. One study revealed bilateral focal hypermetabolism in the caudate, putamen, and anterior cingulum [21]. Jan Novy et al. found a significant increase in FDG uptake in the caudate nuclei in episodes of varying intensity and delay from the onset of the symptoms [32]. Another study identified selective caudate nucleus hypermetabolism along the previously described gradient of activity [32]. In the present study, putamen, caudate, and thalamus were identified as hypermetabolism brain regions with visual analysis. However, only the SUVR of putamen showed a significant statistical difference compared to the control group. The involvement of the cerebellum is rare. Cerebellum involvement is rare. The present study also revealed a case of the involvement of the left cerebellum.

There were several limitations that were associated with our work. First, there may be a potential selection bias toward races and regions, considering that the patients who participated were from a single health center. Second, analyses of subgroups with variable clinical symptoms were not conducted. This calls for further investigation using a larger sample size to elucidate the metabolic alterations of the brain, with different neuropsychiatric symptoms. Third, the current study’s sample size is insufficient for a useful comparison of MR images to determine variations between disease and control groups using machine learning methods, as well as a comparison between patterns of FDG PET and different T2-FLAIR features, which necessitate a further accumulation of more cases analyzed in the future assisting resolve this clinical difficulty.

## Conclusions

In conclusion, anteroposterior glucose metabolism gradient (frontal-temporal/parietal-occipital) is a typical pattern of anti-NMDAR encephalitis in the acute and subacute phases, which is also commonly demonstrated in the parietal lobe and cingular cortex with hypermetabolism of anterior portion and hypometabolism of posterior portion. This may be a potential indicator for the diagnosis of this disorder. In addition, MRI is an important and reliable neuroimaging modality to assist in the correct evaluation of activity on ^18^F-FDG PET.

## Data Availability

The datasets used and analyzed during the current study are available from the corresponding author on reasonable request.
